# Gamma delta T cells in hepatocellular carcinoma: Sunrise of new therapy based on Vδ2 T cells?

**DOI:** 10.1002/ctm2.834

**Published:** 2022-04-22

**Authors:** Zuzana Macek Jilkova

**Affiliations:** ^1^ Université Grenoble Alpes Grenoble France; ^2^ Institute for Advanced Biosciences Research Center UGA/Inserm U 1209/CNRS 5309 La Tronche France; ^3^ Service d'hépato‐gastroentérologie Pôle Digidune CHU Grenoble Alpes La Tronche France

**Keywords:** Hepatocellular carcinoma, tumour microenvironment, γδ T cells

1

The past decade has been marked by a major evolution in the understanding of how hepatocellular carcinoma (HCC) develops and evolves, highlighting the role of the immune system and the particular immune subpopulations infiltrating the liver. Immunotherapies such as immune checkpoint inhibitors have changed the landscape of HCC treatment. However, despite initial enthusiasm, the proportion of responders to immune checkpoint inhibitors remains low. Recent data show that approximately 70% of HCC patients treated with new combination immunotherapies do not respond satisfactorily to the treatment, and their survival prognosis remains critically poor.^1^, ^2^ Therefore, other immunotherapeutic strategies, including immune cell‐based strategies, have been intensively studied to improve an efficient antitumour immune response.

Gamma delta T cells (γδ T cells) are nonconventional T lymphocytes, with a T‐cell receptor composed of a gamma and a delta chain. Depending on the T‐cell receptor (TCR) structure, γδ T cells can be divided into several subtypes. The main subpopulations in humans are Vδ1 and Vδ2 T cells. Vδ1 cells are found mostly in tissues, while Vδ2 T cells are the main subtype of γδ T cells in circulation.^3^ γδ T cells are involved in tumour immune surveillance and can be attractive effector cells for cancer immunotherapy due to their major histocompatibility complex (MHC)‐independent reactivity and independence from cancer neoantigens. These characteristics are especially advantageous for the treatment of HCC, which is known for its highly heterogeneous tumour microenvironment. However, little is known about the phenotype, activity, and metabolic status of γδ T cells and their subsets in HCC patients at the single‐cell level. A new study by Wenjing He, Yi Hu et al. 2022^4^ revealed alterations in the effector functions and metabolic changes of γδ T cells in HCC patients compared to healthy liver tissues. The authors observed that although γδ T cells from HCC livers retain cytotoxicity against cancer cells, they display exhausted gene expression patterns, upregulated mitophagy pathways, alterations in metabolism with enriched glutamine metabolism, downregulation of oxidative phosphorylation, and modifications of other pathways. Further experiments indicated a drastic loss of TCR diversity in intratumoural γδ T cells from HCC patients and a shift in the Vδ1 to Vδ2 T‐cell subpopulation in HCC liver tissue. Next, the authors observed that LAG3 is the main inhibitory immune checkpoint upregulated in γδ T cells from HCC patients, which was associated with glutamine deficiency.

The authors proposed that the transfer therapy of allogeneic Vδ2+ γδ T cells, expanded from peripheral blood mononuclear cells of healthy donors, could complement the functional loss observed in intratumoral γδ T cells and serve as a new treatment strategy in HCC patients, especially when combined with LAG3 inhibitor. These findings now require validation through larger cohort studies, including different aetiological HCC subsets, as the viral‐HCC, investigated by He et al., is characterised by a unique immune microenvironment and may trigger different immune responses compared to HCC with NASH origin.^5^

The improvement in the understanding of HCC infiltrating γδ T cells, including the recent characterisation of tissue‐resident γδ T cells,^6^ provides an important rationale for the development of γδ T‐cell‐based immunotherapy and can help move the field forward and efficiently translate recent findings to more effective HCC treatments.
